# Toxic Effects of Industrial Flocculants Addition on Bioconversion of Black Soldier Fly Larvae (*Hermetia illucens* L.)

**DOI:** 10.3390/insects13080683

**Published:** 2022-07-28

**Authors:** Zhaochang Zhang, Liqi Chen, Kunlun Yang, Tao Wang, Yuting Wang, Yifan Jia, Yijiang Yin, Peng Gu, Hengfeng Miao

**Affiliations:** 1 School of Environmental and Civil Engineering, Jiangnan University, Wuxi 214122, China; 6201405096@stu.jiangnan.edu.cn (Z.Z.); 6211405042@stu.jiangnan.edu.cn (L.C.); yangkunlun@jiangnan.edu.cn (K.Y.); 6211405029@stu.jiangnan.edu.cn (Y.W.); 6201403007@stu.jiangnan.edu.cn (Y.J.); 6201405088@stu.jiangnan.edu.cn (Y.Y.); 2 Jiangsu Engineering Laboratory of Biomass Energy and Carbon Reduction Technology, Jiangnan University, Wuxi 214122, China; 3 School of Environment Engineering, Wuxi University, Wuxi 214105, China; wangtao0532@163.com

**Keywords:** resource utilization, black soldier fly larvae, poly aluminum chloride, insect bioconversion

## Abstract

**Simple Summary:**

The black soldier fly (BSF) is a saprophagous insect that has been applied to organic waste management while providing high-quality insect protein. Flocculants are widely present in organic wastes that could be treated by black soldier fly larvae (BSFL), such as food wastes, municipal sludge, and cyanobacteria sludge. This study investigated the effect of flocculants on bioconversion of BSFL. The results showed that the addition of flocculant affected the bioconversion efficiency and nutritional composition of BSFL. The relative abundance of bacterial genera related to lipid metabolism decreased with increasing flocculant concentrations while disease-related taxa increased in relative abundance. This study could serve as a reference for related research and applications in the future.

**Abstract:**

Black soldier fly is a saprophagous insect that has been widely reported in recent years due to its excellent performance in bioremediation. Due to the widespread presence of flocculants in the organic waste treated by black soldier fly larvae, this study aimed to evaluate the potential impacts and risks of flocculant addition (a combination of poly aluminum chloride and polyacrylamide with the ratio of 50:1). Results showed that the growth and weight of BSFL in the high-exposure groups (≥200 mg/L) were inhibited. The bioaccumulation of aluminum (Al) in larvae was estimated, and the proportions of different Al forms in the frass from high to low were the residual state (41.38% to 67.92%), water-soluble state (16.88% to 37.03%), acid-soluble state (8.45% to 18.72%), and alkali-soluble state (3.38% to 5.14%). The relative abundance of bacterial genera related to lipid metabolism decreased with increasing flocculant concentrations while disease-related taxa increased in relative abundance. The results serve as a reference for subsequent research and application of the treatment of flocculant-contaminated waste by BSFL.

## 1. Introduction

With increased urbanization and industrialization, large amounts of organic waste have seriously affected the ecological quality and resulted in a lot of environmental problems [1]. In addition to traditional technologies, saprophagous insects have been extensively investigated to treat organic waste in recent years. The black soldier fly is a typical saprophagous insect originating from the Americas, and its larvae have been used in the bioremediation of organic waste [2]. Apart from food waste [3], black soldier fly larvae (BSFL) can also be used to treat various animal manures [4], cyanobacterial mud [5], and municipal sludge [6]. As a clean and environmentally friendly technology, BSFL can not only efficiently reduce the amount of organic waste but also provide high-quality insect protein with good economic benefits.

Many factors can affect the bioconversion efficiency of BSFL, including temperature, humidity, substrate nutrients, and additives in the substrate [7]. Identifying the influence of these factors is instructive for the application of this technique. In the actual production process, some flocculants, such as poly aluminum chloride (PAC), polyacrylamide (PAM), and polyaluminum sulfate (PAS), are added to cyanobacterial mud, municipal sludge, and food production wastes due to the practical needs of solid-liquid separation and volume reduction. Taking the cyanobacterial mud of Taihu Lake in China as an example, 425 mg/L of flocculant (a combination of PAC and PAM with the ratio of 50:1) was added to the harvested cyanobacteria to dehydrate it into cyanobacterial mud. As a result, a large quantity of flocculants can be detected in the organic wastes for BSFL treatment, and it is crucial to estimate the effect of these flocculants on the bioconversion of BSFL and the quality of the final product.

Flocculants are usually divided into inorganic and organic flocculants. Inorganic flocculants mainly include the iron and aluminum (Al) preparation series. The accumulation of metal ions in BSFL has been reported in previous research [8]. These metals accumulate in the BSFL and may spread along the food chain [9], which leads to adverse effects on subsequent resource utilization. Al is a metal that widely exists in nature and has neurotoxicity [10] and skeletal toxicity to humans [11]. Different from the previous conclusion [12], the accumulation of Al in BSFL was found in this study and the accumulated Al in BSFL increased with the increase in the Al addition. Due to the application of BSFL and their frass in agriculture, animal husbandry, and food industry, Al may be accumulated and transferred along the food chain, eventually threatening human health. However, the mechanism of the bioaccumulation of Al in BSFL and the form of Al in frass remains unclear.

Gut microbes play an important role in insect growth and development, and the host insects are mainly affected in the following ways [13]: (1) synthesizing nutrients, such as essential amino acids; (2) directly or indirectly modulating the host’s immune system against stress; and (3) producing active substances to regulate host behavior, such as reproduction, mating, and gathering. Gut microbial species are highly dependent on the external environment, food, and gut environment. Changes in the environment and food may affect the microbial community and metabolic function [14], which, in turn, may affect the growth of the host [15]. Therefore, it is necessary to clarify the effect of flocculants on the microbial communities and functions in the gut of BSFL.

In summary, flocculants are widely present in organic wastes treated by BSFL; however, their effects on the bioconversion of BSFL remain unclear. Our research aimed to evaluate the potential impacts and risks of flocculants on the bioconversion of BSFL by studying the development of larvae, larval nutritional components, Al bioaccumulation, different forms of Al in frass, and changes in the microbial communities and functions. This study provides a basis for research on the treatment of flocculant-contaminated waste by BSFL. In addition, these results can guide the subsequent resource utilization of larvae and frass.

## 2. Materials and Methods

### 2.1. Black Soldier Fly Eggs and Feed Substrates

Black soldier fly eggs (Yuwanke Company, Wuxi, China) were hatched in wheat bran with 50% moisture content, 30 °C, and 60% humidity. In order to reduce the error, wheat bran purchased from a local store (Wuxi, China) was dried to a constant weight at 60 °C and compounded with water to form the substrate according to the requirements.

The flocculants PAC and PAM were obtained from Wuxi Municipal Algae-Water Separation Station, with a mass ratio of 50:1. The main composition of industrial PAC is Al_2_O_3_, accounting for about 27–30%. Its raw materials include calcium aluminate, hydrochloric acid, bauxite, and kaolin. The main component of industrial PAM is polyacrylamide, which contains a small amount of acrylamide monomer. For practical application, the concentration of flocculant added was 425 mg/L algae water, and the Al content in the produced algal mud was 148.89 mg/kg dry matter. Therefore, the experimental groups were set up according to the results mentioned above, and the flocculant solution was mixed with the wheat bran.

### 2.2. Experimental Design

Experiments were run at 30 °C and 70% humidity. First, 8-day-old BSFL were added to 50 g of wheat bran with a density of 5 larvae/g substrate (dry weight). The moisture content of the wheat bran was 70%.

Six experimental groups were set up in this study. The feed groups of 0, 50, 100, 200, 300, and 400 mg/L mixed flocculant were named Z1, Z2, Z3, Z4, Z5, and Z6, respectively. Each group was repeated three times. In the local municipal algae-water separation station, the dosage of flocculant was 425 mg/L. The moisture content of the harvested cyanobacteria mud was about 90%, and it needed to be compounded with other feeds to achieve suitable conditions for BSFL. Therefore, the concentration used in this experiment was lower than 425 mg/L.

The experiment ended when the first pupae appeared in each group. The larvae and feces were separated and frozen in a −80 °C freezer.

### 2.3. Parameters of BSFL Growth

The development day refers to the time from successful hatching to the emergence of the first pupae. Larvae were dried at 60 °C for 2 days to obtain dry matter. In each group, a certain number of dried larvae were randomly selected and weighed to calculate the average larval dry weight. The bioconversion rate refers to the ratio of the dry weight of larval biomass to the weight of the substrate added.

### 2.4. Determination of Nutrients

Crude protein (CPRO), crude fat (CFAT), and crude ash (CASH) were measured using the Kjeldahl method, Soxhlet extraction method, and combustion weighing method, respectively.

CPRO: First, 0.5 g dried sample was accurately weighed into a digestion tube. After the addition of 0.2 g of copper sulfate, 3 g of potassium sulfate, and 10 mL of concentrated sulfuric acid, the mixture was digested in a digest furnace. The distillation process was carried out on a Kjeldahl nitrogen determinator (Distillation Unit K-350, BUCHI, Flawil, Switzerland). After distillation, the sample containing indicator (methyl red and bromocresol green) was titrated to red with 0.05 M hydrochloric acid. A carbon-nitrogen conversion factor of 4.76 was selected to calculate the crude protein [16].

CFAT: BSFL powder was extracted with anhydrous ether at 60 °C for 8 h in a Soxhlet extractor. Finally, the solvent was recovered, and the beaker was dried and weighed to obtain the crude fat weight.

CASH: In total, 2 g dried larvae was accurately weighed and burnt to a constant weight in a muffle furnace at 550 °C.

The amino acids measured in this study were hydrolyzed amino acids. In total, 200 mg dried insect powder and 8 mL 6 M hydrochloric acid were successively added into the hydrolysis tube, and nitrogen gas was flushed for 3 min. The samples were hydrolyzed at 120 °C for 22 h. All the hydrolyzed samples were transferred to test tubes and neutralized with 4.8 mL 10 M NaOH. After the samples were diluted to 25 mL with distilled water, they were filtered and centrifuged. Finally, 400 μL supernatant was taken in a liquid vial and determined by high-performance liquid chromatography (Agilent1260, Agilent, Waldbronn, Germany).

The determination of fatty acids followed El-Dakar’s method [17]. Briefly, the crude fat collected after Soxhlet extraction was heated to dissolve it with 2 mL 0.5 mol/L NaOH methanol solution at 60 °C in a water bath. After cooling, 2 mL 25% boron trifluoride ether solution was added, and the mixture was esterified at 60 °C for 30 min. Then, 2 mL normal hexane and 2 mL saturated sodium chloride solution were successively added and shaken. After centrifugation, the upper organic phase was transferred to a liquid vial and a small amount of sewage sodium chlorate was added to remove the remaining trace amount of water. The prepared samples were determined by gas chromatography (GC-2030AF, Shimadzu, Kyoto, Japan).

### 2.5. Metal Detection

Al in BSFL: In total, 0.3 g dried sample was weighed into a digestion tube, and 8 mL nitric acid and 2 mL hydrogen peroxide were successively added. The samples were digested in a microwave digestion instrument (Multiwave PRO, Anton Paar, Graz, Austria). The digestion temperature was set to rise to 180 °C in 25 min, held at 180 °C for 40 min, and then dropped to 70 °C. After the samples were heated to remove acid, they were diluted to 10 mL and passed through a 0.22-μm filter. Finally, the Al element was determined using ICP (Optima 8300, PerkinElmer, Waltham, MA, USA).

Forms of Al in frass: Determination of the Al forms in frass referred to the detection methods of heavy metals in soil and compost [18]. The forms of Al measured in this study included water-soluble fraction, acid-soluble fraction, alkali-soluble fraction, and residual fraction. The air-dried frass was ground into a powder and sieved using a 1-mm sieve. (1) In total, 1.5 g frass was stirred with 12 mL deionized water at room temperature for 30 min. After the solution was centrifuged at 6000 rpm for 10 min, the supernatant was passed through a 0.45-μm filter to obtain the water-soluble fraction. (2) After rinsing with deionized water, 1.5 g frass was stirred for 2 h by 12 mL 0.15 mol/L NaOH and 12 mL 0.1 mol/L HCl, respectively, and the remaining steps were the same as above to obtain alkali-soluble fraction and acid-soluble fraction. (3) After rinsing, 1.5 g frass was digested by a microwave digester using 2 mL nitric acid, 6 mL hydrochloric acid, and 2 mL hydrofluoric acid to obtain the residual fraction. The Al detection method referred to the above detection of Al in BSFL.

### 2.6. S RNA Sequencing

After the reaction, a certain number of larvae were taken from each group for surface disinfection with absolute ethanol. Two grams of the dissected sample were sent to Majorbio (Shanghai, China) for DNA extraction. Total microbial genomic DNA was extracted from samples using the DNeasy^®^ PowerSoil^®^ Pro Kit (QIAGEN, Hilden, Germany). The quality and concentration of DNA were determined by 1.0% agarose gel electrophoresis and a NanoDrop^®^ ND-2000 spectrophotometer (Thermo Scientific Inc., Waltham, MA, USA). The primers amplified by PCR were 338F primer 5′-ACTCCTACGGGGGGGCAGCAG-3′ and 806R primer 5′-GGACTAChVGGGTWTCTAAT-3′ [19]. Purified amplicons were pooled in equimolar amounts and paired-end sequenced on an Illumina MiSeq PE300 platform/NovaSeq PE250 platform (Illumina, San Diego, CA, USA) according to the standard protocols of Majorbio Bio-Pharm Technology Co. Ltd. (Shanghai, China). The sequences were clustered into operational taxonomic units (OTUs) at a similarity level of 97% to generate rarefaction curves and to calculate the richness and diversity indices. The taxonomy of each OTU representative sequence was analyzed by the Ribosomal Database Project Classifier tool.

### 2.7. Data Analysis

The results were analyzed by one-way ANOVA using SPSS 23 (SPSS Inc., Chicago, IL, USA). Post-hoc multiple comparisons were determined by the use of LSD and Duncan test. *p* < 0.05 was considered a marker of a significant difference in the comparison values. The data analysis and data visualization of microorganisms were carried out on the Majorbio cloud platform (https://cloud.majorbio.com; (accessed on 15 May 2022)). Based on the OTUs information, rarefaction curves and alpha diversity indices, including the observed OTUs, Shannon, Chao1, and Simpson indices, were calculated with Mothur v1.30.1. The metagenomic functions of the OTUs were predicted by PICRUSt (Phylogenetic Investigation of Communities by Reconstruction of Unobserved States) [20] and the Kyoto Encyclopedia of Genes and Genomes (KEGG) database [21].

## 3. Results and Discussion

### 3.1. Indicators of BFSL Bioconversion and Product Quality

#### 3.1.1. BFSL Bioconversion Performance

The bioconversion performance indicators in this study mainly referred to the development days, larval survival rate, larval weight, dry mass reduction, and bioconversion rate.

Table 1 indicates that the flocculant addition had little effect on the survival rate of larvae and the reduction rate of substrate, which were 94.27–98.00% and 55.39–60.01%, respectively. Meanwhile, the development days, BSFL weight, and bioconversion rate showed the same trend. When the amount of flocculant added was 0–100 mg/L, the development days were maintained at 17.50 days; for an addition amount greater than 100 mg/L, the development days of BSFL were prolonged, and finally reached 19.00 days. The weight of the larvae did not change much for a flocculant dosage of 0–200 mg/L; when the dosage was more than 200 mg/L, the weight of the larvae decreased significantly (*p* < 0.05), and finally dropped to 13.99 ± 0.51 mg. The bioconversion rate reflected the ability of the unit substrate to be converted into BSFL biomass. When the flocculant dosage was 0–200 mg/L, the bioconversion rate was less affected by the flocculant and maintained at 8.42–9.17%; when the addition amount was 300–400 mg/L, the bioconversion rate dropped from 7.80% to 6.71%.

From the perspective of bioconversion performance, exposure to low concentrations (≤100 mg/kg) of flocculants had only a slight effect on BSFL, and some indicators showed no differences. For high-exposure groups, the development days of BSFL were prolonged (18.10–19.00 days), the larval weight was reduced (18.56–13.99 mg), and the bioconversion rate was decreased (8.42–6.71%); however, the larval survival rate and substrate reduction rate were still less affected. Overall, the flocculants PAC and PAM induced unfavorable effects on bioconversion of BSFL at higher concentrations. In future applications, organic waste containing flocculants can be compounded with other feeds to effectively reduce its impact.

#### 3.1.2. Nutrient Content of BFSL

The nutrients of BSFL are mainly protein and fat [22]. With the increase in the flocculant, the CPRO content increased first and then decreased, reaching a peak of 42.83% at 200 mg/L. The CPRO content of BSFL exposed to high concentrations of flocculant was slightly higher than that of larvae exposed to low concentrations. The content of CFAT decreased from 32.91% to 28.17% with the increase in the flocculant concentration. The crude ash increased from 6.98% to 9.91% with the increase in the flocculant concentration, which might be related to the accumulation of Al in the larvae.

**Figure 1:**
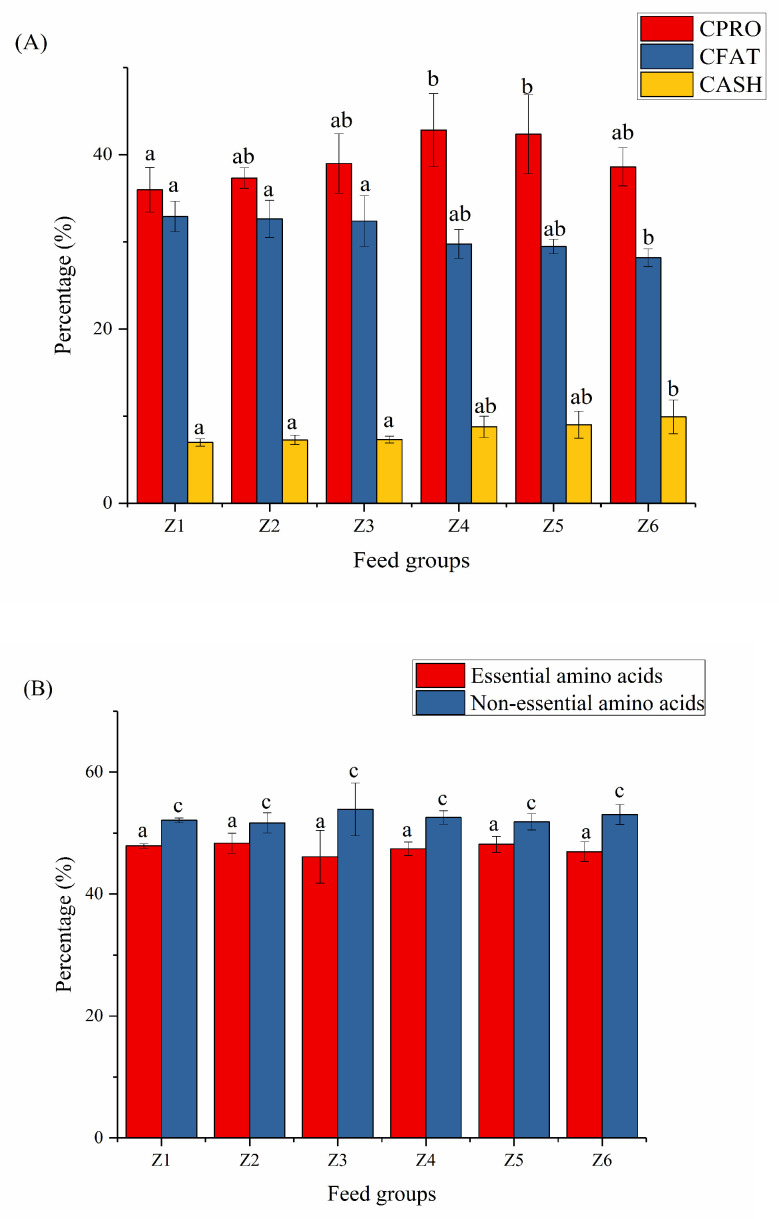

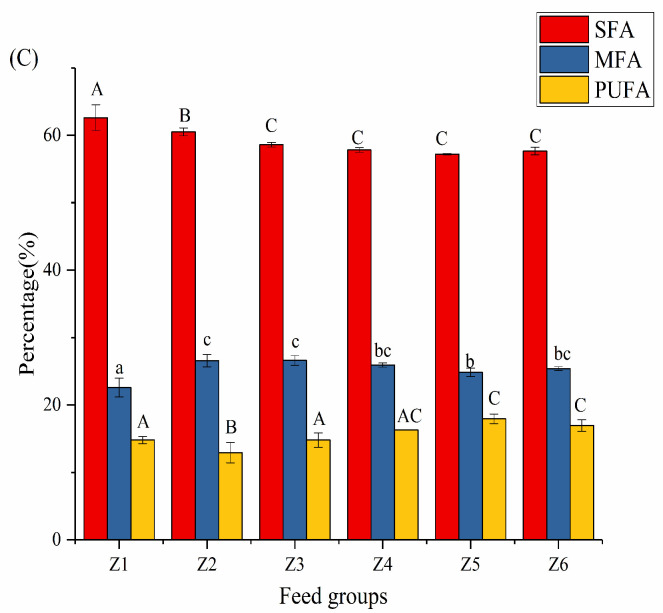
Nutrient composition of BSFL. (**A**) Nutrition, (**B**) amino acids, and (**C**) fatty acids. Different characters indicate significant difference (*p* < 0.05). Z1, Z2, Z3, Z4, Z5 and Z6 represented the six experimental groups of 0, 50, 100, 200, 300 and 400 mg/L mixed flocculant, respectively.

Amino acids are the basic units that make up protein [23]. Amino acids are essential nutrients for animal growth, which are divided into essential amino acids and non-essential amino acids [24]. Figure 1 shows that with the increase in flocculants, the proportion of essential amino acids and non-essential amino acids in BSFL did not change significantly (*p* > 0.05). Essential amino acids were less than non-essential amino acids. Table S1 shows that each amino acid was not significantly affected by the concentration of flocculant (*p* > 0.05). Previous reports found that different substrates had little effect on the amino acids in BSFL [25]. This study confirmed that the combination of PAC and PAM has no effect on the amino acid composition of BSFL.

Fats are made up of fatty acids and glycerol. Fatty acids are divided into saturated fatty acids (SFAs), monounsaturated fatty acids (MFAs), and polyunsaturated fatty acids (PUFAs). Among them, monounsaturated fatty acids, such as omega-7 [26], and polyunsaturated fatty acids, such as omega-3 [27] and DHA [28], have been widely reported in recent years, which are very beneficial to human growth and development. In this study, saturated fatty acids accounted for the highest proportion, followed by monounsaturated fatty acids while polyunsaturated fatty acids accounted for the lowest. The proportion of fatty acids may be related to the choice of feed. Eating different feeds may lead to the accumulation of different fatty acids [24]. With the increase in the flocculant concentration, the proportion of saturated fatty acids first decreased slightly, and then remained at a certain level. Statistically, both monounsaturated and polyunsaturated fatty acids were less affected by the flocculant, with slight fluctuations at lower concentrations.

### 3.2. Bioaccumulation and Forms of Al

#### 3.2.1. Al Bioaccumulation in BSFL

Al is one of the most abundant metals on Earth and is neurotoxic, skeletal, and hemotoxic [29], and PAC is widely used as a cheap and efficient flocculant in industry. In this study, the accumulation of Al in BSFL was discovered.

**Figure 2:**
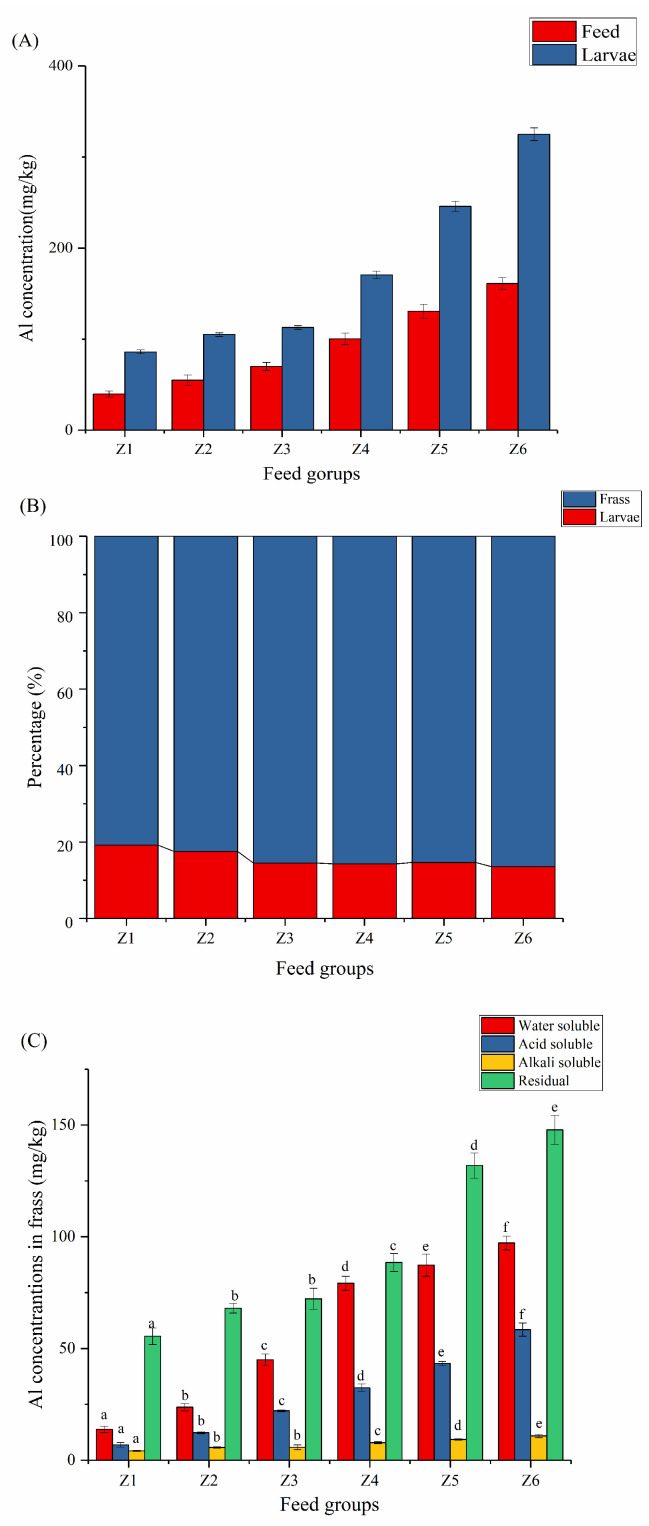
Bioaccumulation of Al in BSFL (**A**), distribution of Al in larvae and frass (**B**), and existing forms of Al (**C**). Different characters indicate a significant difference (*p* < 0.05). Z1, Z2, Z3, Z4, Z5, and Z6 represent the 6 experimental groups of 0, 50, 100, 200, 300, and 400 mg/L mixed flocculant, respectively.

Figure 2A shows the concentration of Al present in the substrate and accumulated in the larvae. Due to the inherent presence of Al in wheat bran, the Al in the substrate was calculated as an integrated value. When the flocculant concentration increased from 0 to 400 mg/L, the Al accumulated in the BSFL increased from 80.00 to 325.01 mg/kg. Figure 2B shows that about 13.54–19.17% of the Al accumulated in the BSFL biomass, and the rest remained in the frass. With the increasing concentration of flocculants, the proportion of Al that accumulated in the BSFL biomass gradually decreased. The results of this study show that the ability of BSFL to accumulate Al was restricted with the increasing concentration of flocculants. Proc’s previous study found no accumulation of Al by comparing Al in BSFL biomass at the beginning and end of development [12]. In this study, the accumulation of Al in BSFL was discovered for the first time. The higher the Al concentration in the substrate, the more Al accumulated in the BSFL. The difference in the conclusions may be due to the different experimental settings. In this experiment, a large amount of Al was added into the feed while in Proc’s experiment, the BSFL were fed with conventional feed and the concentration of the metals was detected before and after feeding.

Exposure to metals can affect the growth of BSFL and cause bioaccumulation of metals in larvae [30]. Researchers found that BSFL can accumulate cadmium, lead, and arsenic from substrates while larval developmental days and total fresh weight may be affected [31]. Another study pointed out that the effects of chromium on BSFL can last for the entire life cycle and considered that these two metals could affect the larval development time and pupation rate [32]. However, few studies have been conducted on the effects of Al on the growth and development of BSFL. The results show that Al in flocculants may have a certain inhibitory effect on the bioconversion of BSFL. Future studies need to clarify whether this inhibition is due to the toxicity of Al or a change in the physical properties due to the addition of flocculants.

The bioaccumulation of Al played an important role in the application of BSFL. Since Al can accumulate in the human body and cause chronic toxicity [33], various countries have successively established food and feed standards to limit Al. The WHO/FAO proposes that the weekly tolerance of Al for humans is 2 mg/kg body weight [34], and the Chinese food safety standard GB2760-2014 stipulates that Al residue should be ≤100 mg/kg. The result showed that the accumulation of Al in BSFL cannot be ignored, which may limit the application of BSFL in the food field. China’s current feed hygiene standard GB 13078-2017 has no relevant standards for Al. This means that BSFL containing Al have more application space in animal husbandry and fishery. However, the effects of ingesting Al-contaminated diets on animals and the subsequent environmental and health risks must be considered.

#### 3.2.2. Different Forms of Al in Frass

The main resource utilization way of frass is as organic fertilizer, so the existing form of metal in frass is very important. The existing forms of metals in animal manures are mainly related to the chemical properties of metals and the characteristics of frass [35]. At present, there is no unified and recognized standard to evaluate the existing forms of metals in frass. This study mainly referred to the related research on soil [36]. The water-soluble state mainly includes the dissolved state and low molecular organic matter combined state; the acid-soluble state mainly includes the fulvic acid combined state and adsorption state; the alkali-soluble state is mainly composed of the humic acid complex state; and the residual state is mainly insoluble minerals.

Figure 2C shows that all the forms of Al were positively correlated with the concentration of flocculants. The concentrations of residual Al after bioconversion were 55.46–147.85 mg/kg, accounting for the largest proportion (41.38% to 67.92%). This is consistent with previously reported conclusions [37]. Among them, the concentrations of residual Al in Z1 and Z2 were 147.85 and 131.88 mg/kg, respectively, higher than the other groups (55.46–79.21 mg/kg). Water-soluble Al in manure can be directly absorbed by crops, and its concentrations in the frass were 13.78–97.23 mg/kg (16.88–37.03%). The concentrations of the acid-soluble state and alkali-soluble state were relatively small, which were 6.90–58.45 (8.45–18.72%) and 4.20–10.85 mg/kg (3.38–5.14%), respectively. In fertilizers, the water-soluble and acid-soluble states are more easily utilized while the alkali-soluble and residual states are “inert”. Due to differences in the pH, Al concentration, and existing forms, Al may have stimulatory or inhibitory effects on crops [38]. Therefore, these studies have an important guiding role for the subsequent resource utilization of frass.

### 3.3. Effects on the Gut Microbiota

#### 3.3.1. Effects on the Microbial Community

**Figure 3:**
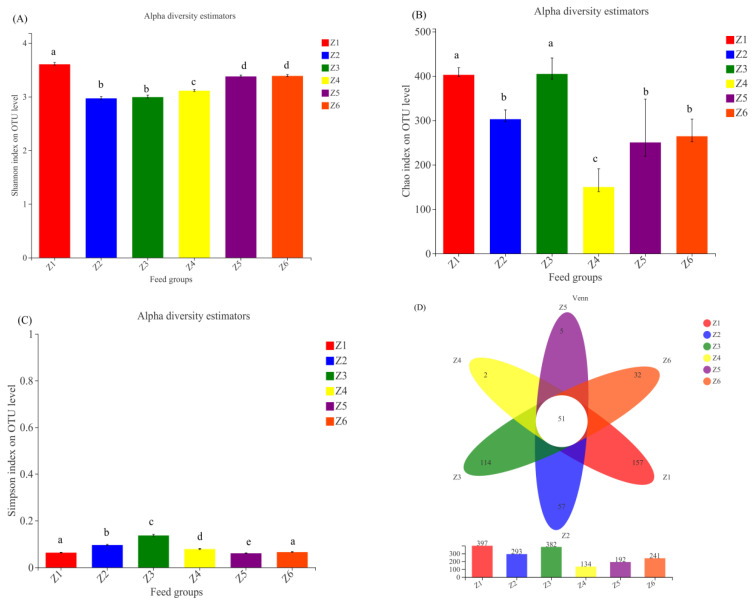
Alpha diversity (**A**): Shannon index, (**B**): Chao1 index, (**C**): Simpson index), and Venn diagram of the obtained OTUs (**D**). Different characters indicate a significant difference (*p* < 0.05). Z1, Z2, Z3, Z4, Z5, and Z6 represent the 6 experimental groups of 0, 50, 100, 200, 300, and 400 mg/L mixed flocculant, respectively.

According to the amplification results of 16S RNA, the OTU richness of low-exposure groups (293–397) was higher than that of high-exposure groups (134–241), indicating that high concentrations of flocculants can affect the species richness of gut microbes in BSFL. The effect of the flocculant addition on the alpha diversity of BSFL gut microbes was analyzed by comparing the Shannon, Chao1, and Simpson indices (Figure 3). A higher Shannon index reflects a higher microbial diversity of the sample while the Simpson index shows the opposite trend. With the increase in the flocculant concentration, the Shannon index first decreased from 3.60 to 2.96 and then rebounded to 3.39. At the same time, the Simpson index first increased from 0.06 to 0.14 and then decreased to 0.07, reaching the peak in the Z3 group. This indicated that the diversity of the gut microbial communities in BSFL decreased first and then increased with increasing flocculant concentration. For the Chao1 index, the value of the low-exposure groups (302.69–404.56) was higher than that of the high-exposure groups (149.83–264.15). This implies that with the addition of flocculant, the abundance of the gut microbes of BSFL decreased.

In this study, the intestinal microorganisms of different groups of BSFL were profiled at the phylum and genus levels. After the addition of flocculants, the community structure of the intestinal microorganisms of BSFL changed significantly. At the phylum level, *Firmicutes*, *Bacteroidetes*, *Actinomycetes*, and *Proteobacteria* were the dominant phylum regardless of the treatment group. The largest proportion was accounted for by *Firmicutes*, which is related to lipid metabolism in mammals [39]. In general, the relative abundance of *Firmicutes* from the low-exposure groups was higher than that from the high-exposure groups, which might be related to the relative decline in crude fat in BSFL. A similar trend was observed in *Bacteroidetes*, another phylum associated with lipid metabolism [40]. The relative abundance of *Proteobacteria* first decreased and then increased with the increase in the flocculant concentration. Since it is seen as a marker of microbial dysbiosis in insects [41], this suggests that low-concentration flocculant exposure may help alleviate gut microbial dysbiosis.

**Figure 4:**
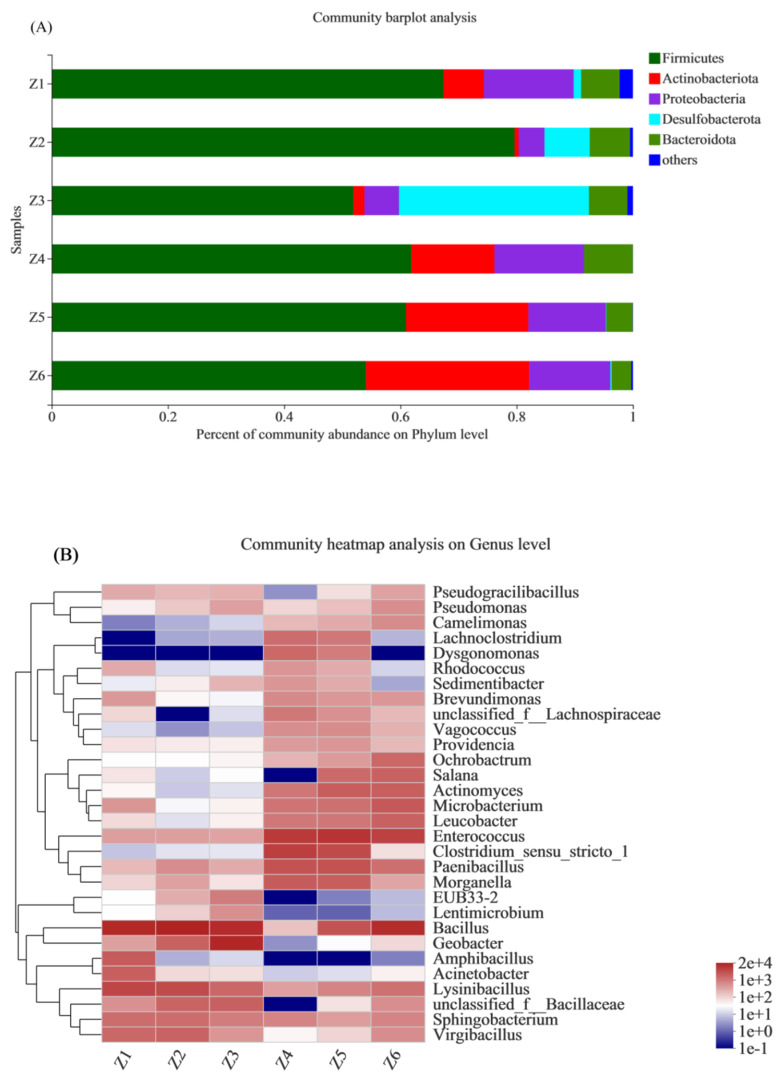
Microbial community at the (**A**) phylum and (**B**) genus levels. Z1, Z2, Z3, Z4, Z5, and Z6 represent the 6 experimental groups of 0, 50, 100, 200, 300, and 400 mg/L mixed flocculant, respectively.

Figure 4B shows a genus-level community heatmap for the top 30 abundances. From the genus level, with the increase in the flocculant concentration, the composition of the intestinal microbial population of BSFL changed significantly. Among the genera listed, the genera related to biological development were basically related to disease. Although *Bacillus* can promote the growth of plants [42], it is also reported to be a widely distributed pathogen to mammalians [43]. The relative abundance of *Enterococcus* increased with the increasing flocculant concentration, and it is thought to be associated with disease in poultry [44] and humans [45]. *Actinomyces* have been reported to be associated with diseases in humans [46]. The abundance of *Actinomyces* in the low-exposure groups was lower than that in the high-exposure groups. This trend suggests that the flocculant addition might cause diseases in BSFL and thus affect the growth. *Brevundimonas* have potential pathogenicity to human beings [47], and the relative abundance of the high-exposure group was generally higher than that of the low-exposure group. It is well known that external stresses may affect the growth of organisms by affecting their gut microbes [48]. In this study, the abundance of genera related to disease increased with the increasing flocculant concentration. This may have an impact on the growth and development of BSFL, leading to adverse effects on the bioconversion efficiency.

#### 3.3.2. PICRUSt Metabolism Functions Prediction

**Figure 5:**
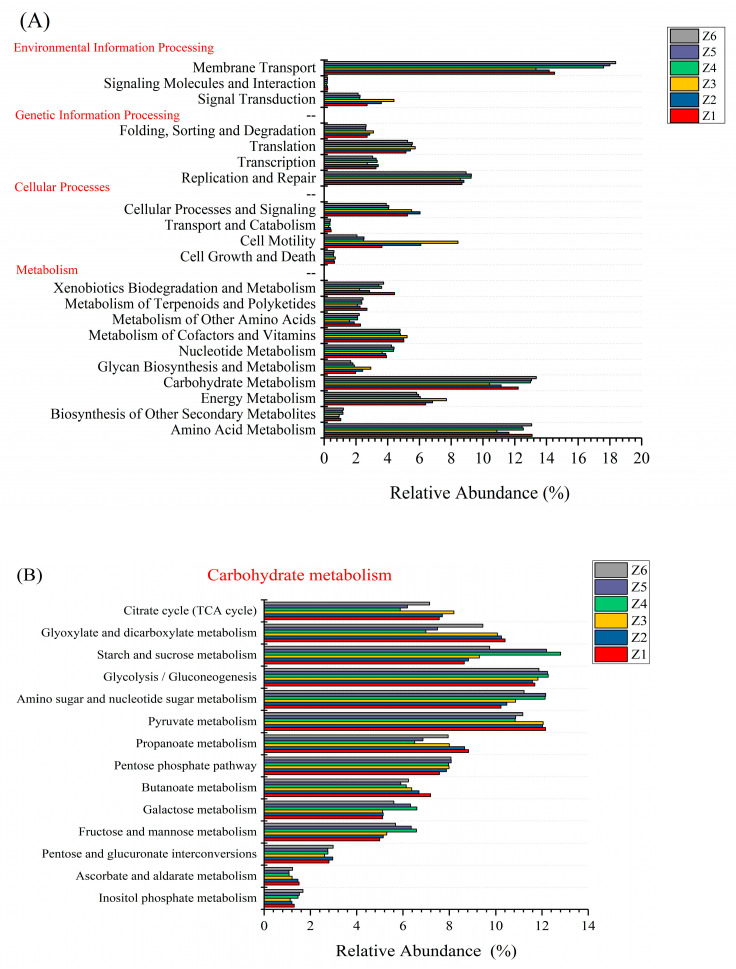

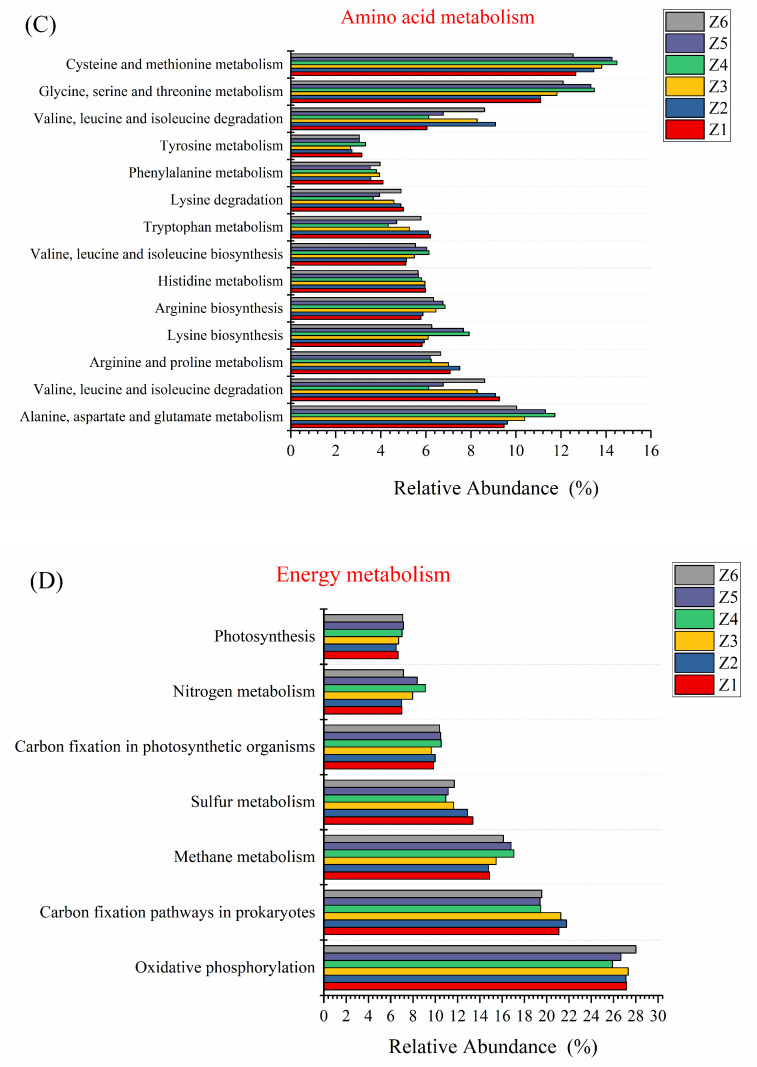
Variation in bacterial function profiles of BSFL at the level 2 (**A**) and level 3 (**B**–**D**) KEGG ortholog function predictions analyzed by PICRUSt. Z1, Z2, Z3, Z4, Z5, and Z6 represent the 6 experimental groups of 0, 50, 100, 200, 300, and 400 mg/L mixed flocculant, respectively.

It is well known that there is a close correlation between intestinal microorganisms and the metabolic ability of the host [49]. Changes in the composition of intestinal microorganisms may lead to changes in the function of intestinal microorganisms. To further understand the effect of flocculant addition on the metabolic potential of the BSFL gut microbes, the microbial functions of the OTUs were predicted using PICRUSt (Figure 5). The protein sequences in the samples were mainly related to environmental information processing, genetic information processing, metabolism, and cellular processes. Among them, the functions related to metabolism mainly included amino acid metabolism, biosynthesis of other secondary metabolites, energy metabolism, carbohydrate metabolism, glycan biosynthesis and metabolism, nucleotide metabolism, metabolism of cofactors and vitamins, metabolism of other amino acids, and metabolism of terpenoids and polyketides at the KEGG pathway level 2 level (Figure 2A). Overall, the relative abundance of carbohydrate metabolism, amino acid metabolism, and energy metabolism changed greatly. The functions of carbohydrate metabolism, amino acid metabolism, and energy metabolism were further predicted and analyzed at the KEGG pathway level 3 level. The functions of carbohydrate metabolism (Figure 5B) and amino acid metabolism (Figure 5C) at level 3 changed considerably. Although, there were changes in energy metabolism at the KEGG pathway level 2. However, from level 3, the relative abundance of each predicted function was less affected by the flocculant (Figure 5D). The results show that the addition of flocculants may have a greater impact on the metabolism of carbohydrates and amino acids but less on energy metabolism. Flocculants may affect the bioconversion performance of BSFL by affecting the metabolic capacity of gut microbes. Since this method can only reflect its metabolic potential, more accurate evidence is needed in the future to demonstrate the effect of flocculants on the metabolic capacity of BSFL.

## 4. Conclusions

This study evaluated the potential impacts and risks of flocculants on the bioconversion of BSFL. Results showed that the bioconversion of BSFL was inhibited by flocculant addition. In this study, the bioaccumulation of aluminum in larvae was estimated, and the proportions of different Al forms in the frass from high to low were the residual state (41.38% to 67.92%), water-soluble state (16.88% to 37.03%), acid-soluble state (8.45% to 18.72%), and alkali-soluble state (3.38% to 5.14%). The relative abundance of bacterial genera related to lipid metabolism decreased with increasing flocculant concentrations while disease-related taxa increased in relative abundance. The results of this study serve as a reference for subsequent research and application of the treatment of flocculant-contaminated waste by BSFL.

## Figures and Tables

**Table 1:** Development days, larval survival rate, dry mass reduction, and feed conversion ratio (dry mass) and the bioconversion (dry mass) of different treatment groups.

Feed Groups	Flocculant Contents (mg/L)	Development Day (Days)	Larval Dry Weight (mg)	Larval Survival Rate (%)	Dry Mass Reduction (%)	Bioconversion Rate (%)
Z1	0	17.50 ± 0.20 ^a^	18.10 ± 0.75 ^cd^	98.00 ± 1.06 ^b^	60.61 ± 0.94 ^b^	8.87 ± 0.45 ^cd^
Z2	50	17.50 ± 0.20 ^a^	18.90 ± 0.34 ^cd^	97.07 ± 1.29 ^ab^	59.67 ± 0.60 ^b^	9.17 ± 0.27 ^d^
Z3	100	17.50 ± 0.20 ^a^	18.56 ± 0.77 ^d^	97.07 ± 1.62 ^ab^	60.69 ± 1.85 ^b^	9.01 ± 0.42 ^d^
Z4	200	18.10 ± 0.20 ^b^	17.86 ± 0.11 ^c^	94.27 ± 1.67 ^a^	59.74 ± 1.38 ^b^	8.42 ± 0.11 ^c^
Z5	300	18.10 ± 0.20 ^b^	16.21 ± 0.26 ^b^	96.27 ± 2.27 ^ab^	59.16 ± 3.15 ^b^	7.80 ± 0.09 ^b^
Z6	400	19.00 ± 0.20 ^c^	13.99 ± 0.51 ^a^	96.00 ± 1.44 ^ab^	55.39 ± 1.38 ^a^	6.71 ± 0.24 ^a^

Data are mean ± standard error; different letters above bars indicate a significant difference (*p* < 0.05) in different indicators in the same treatment group.

## Data Availability

All data generated or analyzed during this study are included or referred to appropriately in this article and its Supplementary Materials file.

## Supplementary Materials

The following supporting information can be downloaded at https://www.mdpi.com/article/10.3390/insects13080683/s1, Table S1: Mean (±SD) amino acids concentration (mg/g) of BSFL according to different feed groups, Table S2: Mean (±SD) fatty acids content (%) of BSFL according to different feed groups.
