# Supplementary material for: Toxic Effects of Industrial Flocculants Addition on Bioconversion of Black Soldier Fly Larvae (Hermetia illucens L.)
Source: Insects. 2022 Jul 28;13(8):683. doi: 10.3390/insects13080683 (PMC9409322; doi:10.3390/insects13080683)
Supplement: Supplementary file 1 [file insects-13-00683-s001.zip › insects-1817155-supplementary.pdf]

Supplementary Material

MAMUSCRIPT TITLE: Toxic effects of industrial flocculants addition on bioconversion of black soldier fly larvae (Hermetia illucens L.)

AUTHORS: Zhaochang Zhang [a], Liqi Chen [a], Kunlun Yang [a, b], Tao Wang [c], Yuting Wang [a], Yifan Jia [a], Yijiang Yin [a], Peng Gu [a, b, *], Hengfeng Miao[a, b, *]

ADDRESS: [a.] School of Environmental and Civil Engineering, Jiangnan University, Wuxi 214122, PR China

[b.] Jiangsu Engineering Laboratory of Biomass Energy and Carbon Reduction Technology, Jiangnan University, Wuxi 214122, PR China

[c.] School of Environment Engineering, Wuxi University, Wuxi 214105, PR China.

Corresponding authors: Peng Gu, e-mail: penggu@jiangnan.edu.cn

Hengfeng Miao, e-mail: hfmiao@jiangnan.edu.cn

NO. OF TABLES: 2

NO. OF FIGURES: 0

NO. OF PAGES: 3

Table S1. Mean (±SD) amino acids concentration (mg/g) of BSFL according to different feed groups.

Amino acids	Feed groups					
	Z1	Z2	Z3	Z4	Z5	Z6
Threonine (THR)	1.63±0.34[a]	1.54±0.22[a]	1.46±0.24[a]	1.44 ±0.25[a]	1.70 ± 0.22[a]	1.53±0.43[a]
Valine (VAL)	3.16±0.30[a]	3.76±0.82[a]	3.16±0.18[a]	3.95±0.29[a]	3.65±0.28[a]	4.02±0.49[a]
Methionine (MET)	1.52±0.51[a]	1.53±0.27[a]	2.04±0.96[a]	1.78±0.15[a]	2.15±0.46[a]	1.69±0.43[a]
Isoleucine (ILE)	1.74±0.19[a]	1.81±0.30[a]	1.71±0.26[a]	1.93±0.38[a]	1.94±0.25[a]	1.78±0.50[a]
Leucine (LEU)	2.25±0.60[a]	2.69±0.46[a]	2.55±0.43[a]	2.88±0.59[a]	2.91±0.40[a]	2.62±0.73[a]
Phenylalanine (PHE)	1.41±0.16[a]	1.48±0.25[a]	1.35±0.24[a]	1.56±0.32[a]	1.52±0.16[a]	1.40±0.36[a]
Lysine (LYS)	2.17±0.26[a]	2.31±0.46[a]	2.13±0.38[a]	2.53±0.63[a]	2.41±0.30[a]	2.19±0.38[a]
Histidine (HIS)	1.26±0.22[a]	1.28±0.20[a]	1.33±0.22[a]	1.43±0.36[a]	1.38±0.05[a]	1.65±0.19[a]
Arginine (ARG)	1.73±0.30[a]	1.61±0.24[a]	1.99±0.10[a]	1.79±0.51[a]	1.76±0.19[a]	1.74±0.55[a]
Aspartic acid (ASP)	2.84±0.38[a]	3.16±0.54[a]	3.30±0.10[a]	3.45±0.75[a]	3.32±0.33[a]	3.17±0.92[a]
Serine (SER)	1.82±0.69[a]	1.48±0.25[a]	1.64±0.04[a]	1.67±0.42[a]	1.63±0.08[a]	1.54±0.46[a]
Glutamic acid (GLU)	4.66±0.67[a]	5.08±0.94[a]	5.48±0.26[a]	5.70±1.32[a]	5.47±0.46[a]	5.51±1.20[a]
Glycine (GLY)	2.36±0.35[a]	2.67±0.45[a]	2.77±0.16[a]	2.88±0.57[a]	2.86±0.24[a]	2.76±0.49[a]
Alanine (ALA)	3.11±0.49[a]	3.40±0.63[a]	3.73±0.15[a]	3.85±0.89[a]	3.73±0.22[a]	3.76±0.53[a]
Tyrosine (TYR)	1.49±0.19[a]	1.59±0.31[a]	1.51±0.08[a]	1.85±0.21[a]	1.68±0.19[a]	1.68±0.20[a]
Proline (PRO)	2.13±0.36[a]	1.93±0.43[a]	2.14±0.16[a]	2.23±0.41[a]	2.25±0.41[a]	2.46±1.15[a]

Different letters represent significant differences ($P < 0.05$).
Essential amino acids include THR, VAL, MET, ILE, LEU, PHE, LYS, HIS, ARG. The rest are non-essential amino acids.

Table S2. Mean (±SD) fatty acids content (%) of BSFL according to different feed groups.

Fatty acids	Feed groups					
	Z1	Z2	Z3	Z4	Z5	Z6
C10:0	1.90±0.44^{a}	1.22±0.37^{b}	1.54±0.13^{ab}	1.38±0.27^{ab}	1.17±0.24^{b}	1.26±0.12^{b}
C12:0	32.61±1.70^{a}	31.62±1.44^{ab}	30.16±0.13^{b}	30.60±0.59^{b}	29.74±0.78^{b}	30.12±0.66^{b}
C14:0	1.92±0.06^{a}	2.16±0.05^{b}	1.55±0.10^{c}	1.31±0.19^{d}	1.39±0.17^{cd}	1.32±0.02^{d}
C15:0	0.37±0.01^{ab}	0.39±0.02^{abc}	0.38±0.05^{ab}	0.50±0.01^{c}	0.49±0.11^{bc}	0.34±0.08^{a}
C16:0	13.11±0.10^{ab}	14.52±3.20^{ab}	15.00±0.02^{b}	13.43±0.49^{ab}	12.16±0.66^{a}	12.15±0.17^{a}
C17:0	1.05±0.01^{a}	0.92±0.30^{a}	1.38±0.56^{a}	1.28±0.48^{a}	1.51±0.50^{a}	1.01±0.01^{a}
C18:0	8.10±0.02^{a}	6.26±0.35^{b}	5.90±1.04^{b}	6.47±0.66^{bc}	7.48±0.33^{ac}	8.20±0.58^{a}
C20:0	1.64±0.02^{a}	1.89±1.27^{a}	1.16±0.05^{a}	1.46±0.36^{a}	1.64±0.05^{a}	1.54±0.14^{a}
C22:0	1.90±0.07^{a}	1.53±0.42^{ab}	1.53±0.14^{ab}	1.40±0.10^{b}	1.62±0.11^{ab}	1.72±0.36^{ab}
C14:1	4.77±0.40^{a}	5.38±1.53^{a}	5.53±0.18^{a}	5.80±0.37^{ab}	7.02±0.17^{b}	6.92±0.31^{b}
C16:1	6.15±1.18^{ad}	8.73±0.61^{b}	8.35±0.63^{bc}	7.23±0.74^{cd}	5.27±0.64^{ae}	4.36±0.51^{e}
C18:1	11.14±0.22^{a}	12.09±1.92^{ab}	12.32±1.20^{ab}	12.51±0.82^{ab}	12.26±0.09^{ab}	13.81±0.63^{b}
C20:1	0.51±0.04^{a}	0.37±0.07^{b}	0.41±0.02^{ab}	0.38±0.03^{b}	0.31±0.10^{b}	0.31±0.08^{b}
C18:2	12.38±0.49^{a}	10.97±0.91^{b}	11.75±0.17^{ab}	13.77±0.58^{c}	15.01±0.10^{c}	13.84±1.27^{c}
C18:3	1.34±0.01^{a}	0.83±0.14^{b}	1.58±0.40^{ac}	1.45±0.23^{a}	1.59±0.26^{ac}	1.95±0.02^{c}
C20:4	1.08±0.04^{a}	1.12±0.65^{a}	1.46±0.51^{a}	1.05±0.38^{a}	1.34±0.55^{a}	1.15±0.44^{a}

Different letters represent significant differences ($P < 0.05$).
